# A simple method for assessing sample sizes in microarray experiments

**DOI:** 10.1186/1471-2105-7-106

**Published:** 2006-03-02

**Authors:** Robert Tibshirani

**Affiliations:** 1 Health Research & Policy, Stanford University, Stanford, CA 94305, USA

## Abstract

**Background:**

In this short article, we discuss a simple method for assessing sample size requirements in microarray experiments.

**Results:**

Our method starts with the output from a permutation-based analysis for a set of pilot data, e.g. from the SAM package. Then for a given hypothesized mean difference and various samples sizes, we estimate the false discovery rate and false negative rate of a list of genes; these are also interpretable as per gene power and type I error. We also discuss application of our method to other kinds of response variables, for example survival outcomes.

**Conclusion:**

Our method seems to be useful for sample size assessment in microarray experiments.

## Background

Assessment of sample sizes for microarray data is a tricky exercise. The data are complex, as are the biological questions that one might try to answer from such data. What assumptions should one make, and what quantities should be provided as output?

There have been a number of recent papers that address this problem. The authors in [2] utilize an ANOVA model and provides power calculations for various alternative models. In [4] a decision-theoretic approach is used and a hierarchical Bayes model. The authors in [8] examine the roles of technical and biological variability, in determining sample size. In [5] it is assumed that the genes are independent and have equal variance, and false discovery rates and sensitivities are reported. The ssize package [7] also assumes that the genes are independent, but uses pilot data to estimate the variance. It focuses on power and type I error. The proposal of [6] assumes independence of genes; the convenient (but unrealistic) case of equal correlation among all genes is also considered.

All of these approaches may have shortcomings, namely the assumption of equal variances or independence of genes (or both). These assumptions are often violated in real microarray data and can have a real impact on sample size calculations.

We avoid these assumptions in our proposal. We start with the output from a permutation-based analysis for a set of pilot data. From this we estimate the standard deviation of each gene, and the overall null distribution of the genes. Then for a given hypothesized mean difference, we estimate the false discovery rate (FDR) and false negative rate (FNR) of a list of genes. Many authors now favor the FDR over the family-wise error rate (FWER) as the appropriate error measure for microarray studies. The latter is the probability of at least one false positive call, given that we expect many false positive calls among thousands of genes, the FWER does not seem to be as relevant.

Since the calculation is based on the gene scores from permutations of the data, the correlation in the genes is accounted for. Use of the permutation distribution avoids parametric assumptions about the distribution of individual genes. And by working with the scores rather than the raw data, we avoid the difficult task of simulating new data from a population having a complicated (and unknown) correlation structure.

We provide interpretation of our results both in terms of FDR and FNR, and in terms power and type I error. Our proposal is implemented in the current version of the SAM package [1].

Our main focus is on microarray experiments for determining which genes are differentially expressed across two different experimental conditions, like treatment versus control. However our approach is also applicable to other settings, for example studies that correlate survival time with gene expression.

We learned of the proposal in [3] from a referee; it was unknown to us at the time that this paper was written. The resampling-based approach in that paper is very close to the one described here. Some differences are a) by shifting the test-statistics rather than the data, our method is applicable beyond the two-sample problem to general settings like survival data, and b) we report not only false discovery rates but also false negative rates in our assessment of sample sizes.

## The proposed method

First we need some definitions. Table 1 summarizes the outcomes of *m *hypothesis tests on a set of *m *genes.

We have FDR = *V/R *and FNR = *T/*(*m - R*), power = *S/m*_1 _and type 1 error = *V/m*_0_. For simplicity, for assessing sample sizes we choose our rule so that the number of genes called significant (*R*) is the same as the number of non-null genes in the population (*m*_1_). This implies that 1 - power = FDR and type I error = FNR. Hence conveniently, the FDR can be interpreted as one minus the power per gene, and similarly for the FNR.

Here are the details of the calculation for the two-class unpaired case (below we indicate changes necessary for other data types). Let *x_ij _*be the expression for gene *i *in sample *j*; *C*_*j *_is the set of indices for the *n*_*j *_samples in group *j*, for *j *= 1 or 2. The two-sample unpaired t-statistic is

di=x¯i2−x¯i1si     (1)
 MathType@MTEF@5@5@+=feaafiart1ev1aaatCvAUfKttLearuWrP9MDH5MBPbIqV92AaeXatLxBI9gBaebbnrfifHhDYfgasaacH8akY=wiFfYdH8Gipec8Eeeu0xXdbba9frFj0=OqFfea0dXdd9vqai=hGuQ8kuc9pgc9s8qqaq=dirpe0xb9q8qiLsFr0=vr0=vr0dc8meaabaqaciaacaGaaeqabaqabeGadaaakeaacqWGKbazdaWgaaWcbaGaemyAaKgabeaakiabg2da9maalaaabaGafmiEaGNbaebadaWgaaWcbaGaemyAaKMaeGOmaidabeaakiabgkHiTiqbdIha4zaaraWaaSbaaSqaaiabdMgaPjabigdaXaqabaaakeaacqWGZbWCdaWgaaWcbaGaemyAaKgabeaaaaGccaWLjaGaaCzcamaabmaabaGaeGymaedacaGLOaGaayzkaaaaaa@4074@

where

si=[(1/n1+1/n2){∑j∈C1(xij−x¯i1)2+∑j∈C2(xij−x¯i2)2}/(n1+n2−2)]1/2
 MathType@MTEF@5@5@+=feaafiart1ev1aaatCvAUfKttLearuWrP9MDH5MBPbIqV92AaeXatLxBI9gBaebbnrfifHhDYfgasaacH8akY=wiFfYdH8Gipec8Eeeu0xXdbba9frFj0=OqFfea0dXdd9vqai=hGuQ8kuc9pgc9s8qqaq=dirpe0xb9q8qiLsFr0=vr0=vr0dc8meaabaqaciaacaGaaeqabaqabeGadaaakeaacqWGZbWCdaWgaaWcbaGaemyAaKgabeaakiabg2da9iabcUfaBjabcIcaOiabigdaXiabc+caViabd6gaUnaaBaaaleaacqaIXaqmaeqaaOGaey4kaSIaeGymaeJaei4la8IaemOBa42aaSbaaSqaaiabikdaYaqabaGccqGGPaqkcqGG7bWEdaaeqbqaaiabcIcaOiabdIha4naaBaaaleaacqWGPbqAcqWGQbGAaeqaaOGaeyOeI0IafmiEaGNbaebadaWgaaWcbaGaemyAaKMaeGymaedabeaakiabcMcaPmaaCaaaleqabaGaeGOmaidaaOGaey4kaSYaaabuaeaacqGGOaakcqWG4baEdaWgaaWcbaGaemyAaKMaemOAaOgabeaakiabgkHiTiqbdIha4zaaraWaaSbaaSqaaiabdMgaPjabikdaYaqabaGccqGGPaqkdaahaaWcbeqaaiabikdaYaaakiabc2ha9jabc+caViabcIcaOiabd6gaUnaaBaaaleaacqaIXaqmaeqaaOGaey4kaSIaemOBa42aaSbaaSqaaiabikdaYaqabaGccqGHsislcqaIYaGmcqGGPaqkcqGGDbqxdaahaaWcbeqaaiabigdaXiabc+caViabikdaYaaaaeaacqWGQbGAcqGHiiIZcqWGdbWqdaWgaaadbaGaeGOmaidabeaaaSqab0GaeyyeIuoaaSqaaiabdQgaQjabgIGiolabdoeadnaaBaaameaacqaIXaqmaeqaaaWcbeqdcqGHris5aaaa@76C3@

Note that this is the gene score used in the SAM method; see the Remark below regarding the exchangeability constant. If *σ*_*i *_is the true within-group standard deviation for gene *i *(assumed to be the same for each group), then *s*_*i *_^2 ^estimates

var⁡(x¯i2−x¯i1)=σi2(1/n1+1/n2)
 MathType@MTEF@5@5@+=feaafiart1ev1aaatCvAUfKttLearuWrP9MDH5MBPbIqV92AaeXatLxBI9gBaebbnrfifHhDYfgasaacH8akY=wiFfYdH8Gipec8Eeeu0xXdbba9frFj0=OqFfea0dXdd9vqai=hGuQ8kuc9pgc9s8qqaq=dirpe0xb9q8qiLsFr0=vr0=vr0dc8meaabaqaciaacaGaaeqabaqabeGadaaakeaacyGG2bGDcqGGHbqycqGGYbGCcqGGOaakcuWG4baEgaqeamaaBaaaleaacqWGPbqAcqaIYaGmaeqaaOGaeyOeI0IafmiEaGNbaebadaWgaaWcbaGaemyAaKMaeGymaedabeaakiabcMcaPiabg2da9GGaciab=n8aZnaaDaaaleaacqWGPbqAaeaacqaIYaGmaaGccqGGOaakcqaIXaqmcqGGVaWlcqWGUbGBdaWgaaWcbaGaeGymaedabeaakiabgUcaRiabigdaXiabc+caViabd6gaUnaaBaaaleaacqaIYaGmaeqaaOGaeiykaKcaaa@4C49@

Hence a shift of *δ *units in one gene for each sample in group 2 causes an average increase in the score *d*_*i *_of δ/(σi1/n1+1/n2)
 MathType@MTEF@5@5@+=feaafiart1ev1aaatCvAUfKttLearuWrP9MDH5MBPbIqV92AaeXatLxBI9gBaebbnrfifHhDYfgasaacH8akY=wiFfYdH8Gipec8Eeeu0xXdbba9frFj0=OqFfea0dXdd9vqai=hGuQ8kuc9pgc9s8qqaq=dirpe0xb9q8qiLsFr0=vr0=vr0dc8meaabaqaciaacaGaaeqabaqabeGadaaakeaaiiGacqWF0oazcqGGVaWlcqGGOaakcqWFdpWCdaWgaaWcbaGaemyAaKgabeaakmaakaaabaGaeGymaeJaei4la8IaemOBa42aaSbaaSqaaiabigdaXaqabaGccqGHRaWkcqaIXaqmcqGGVaWlcqWGUbGBdaWgaaWcbaGaeGOmaidabeaaaeqaaOGaeiykaKcaaa@3DF5@ (we assume that the proportion of samples in groups 1 and 2 remains the same as we vary the sample size).

Starting with some pilot data, this suggests the following procedure for assessing sample sizes:

1. Estimate the null distribution of the scores, and the per gene standard deviation *σ*_*i*_, by randomly permuting the class labels and recomputing the gene scores for the permuted data.

2. For *k *(the number of truly changed genes) running from (say) 10 to *m*/2, do the following:

• Sample a set of *m *scores from the permutation distribution of the scores

• Add δ/(σ^i1/n1+1/n2)
 MathType@MTEF@5@5@+=feaafiart1ev1aaatCvAUfKttLearuWrP9MDH5MBPbIqV92AaeXatLxBI9gBaebbnrfifHhDYfgasaacH8akY=wiFfYdH8Gipec8Eeeu0xXdbba9frFj0=OqFfea0dXdd9vqai=hGuQ8kuc9pgc9s8qqaq=dirpe0xb9q8qiLsFr0=vr0=vr0dc8meaabaqaciaacaGaaeqabaqabeGadaaakeaaiiGacqWF0oazcqGGVaWlcqGGOaakcuWFdpWCgaqcamaaBaaaleaacqWGPbqAaeqaaOWaaOaaaeaacqaIXaqmcqGGVaWlcqWGUbGBdaWgaaWcbaGaeGymaedabeaakiabgUcaRiabigdaXiabc+caViabd6gaUnaaBaaaleaacqaIYaGmaeqaaaqabaGccqGGPaqkaaa@3E05@ in class 2 to a randomly chosen set of *k *of these scores.

• Find the cutpoint *c *equal to the *k*th largest score in absolute value

• Estimate the FDR and FNR of the rule |*d*_*i*_| > *c*. This is straight forward since we know which genes are truly non-null (they are the ones that were incremented above).

3. Repeat Step 2 *B *times and report the median result for each *k*. We also report the 10th and 90th percentiles of the FDR across the *B *permutations.

In our examples we use a relatively small number of repetitions (*B = *20); this makes the procedure fast and gives sufficiently accurate estimates. For the two-sample problem, we typically require pilot data with at least 4 or 5 samples per class.

The results of this process provide information on how the FDR and FNR will improve if the sample size were to be increased. To get an idea of what values of the mean difference *δ *are appropriate or reasonable, one can look at the values x¯i2−x¯i1
 MathType@MTEF@5@5@+=feaafiart1ev1aaatCvAUfKttLearuWrP9MDH5MBPbIqV92AaeXatLxBI9gBaebbnrfifHhDYfgasaacH8akY=wiFfYdH8Gipec8Eeeu0xXdbba9frFj0=OqFfea0dXdd9vqai=hGuQ8kuc9pgc9s8qqaq=dirpe0xb9q8qiLsFr0=vr0=vr0dc8meaabaqaciaacaGaaeqabaqabeGadaaakeaacuWG4baEgaqeamaaBaaaleaacqWGPbqAcqaIYaGmaeqaaOGaeyOeI0IafmiEaGNbaebadaWgaaWcbaGaemyAaKMaeGymaedabeaaaaa@35B5@ among the significant genes in the pilot data.

This approach can be easily applied to other designs and other types of response parameters. For paired data, we take *n*_1_*= n*_2_*= n*/2 (remember *n *is the total sample size). and all of the above recipe remains the same. For one class data var = σi2
 MathType@MTEF@5@5@+=feaafiart1ev1aaatCvAUfKttLearuWrP9MDH5MBPbIqV92AaeXatLxBI9gBaebbnrfifHhDYfgasaacH8akY=wiFfYdH8Gipec8Eeeu0xXdbba9frFj0=OqFfea0dXdd9vqai=hGuQ8kuc9pgc9s8qqaq=dirpe0xb9q8qiLsFr0=vr0=vr0dc8meaabaqaciaacaGaaeqabaqabeGadaaakeaaiiGacqWFdpWCdaqhaaWcbaGaemyAaKgabaGaeGOmaidaaaaa@30F0@*/n*.

For survival data and Cox's proportional hazards model, the analogue of the mean difference between groups is the numerator of the partial likelihood score statistic, which we denote by *r*_*i*_. Hence we define the gene-specific variance σi2
 MathType@MTEF@5@5@+=feaafiart1ev1aaatCvAUfKttLearuWrP9MDH5MBPbIqV92AaeXatLxBI9gBaebbnrfifHhDYfgasaacH8akY=wiFfYdH8Gipec8Eeeu0xXdbba9frFj0=OqFfea0dXdd9vqai=hGuQ8kuc9pgc9s8qqaq=dirpe0xb9q8qiLsFr0=vr0=vr0dc8meaabaqaciaacaGaaeqabaqabeGadaaakeaaiiGacqWFdpWCdaqhaaWcbaGaemyAaKgabaGaeGOmaidaaaaa@30F0@ via the relation var (*r*_*i*_) = σi2
 MathType@MTEF@5@5@+=feaafiart1ev1aaatCvAUfKttLearuWrP9MDH5MBPbIqV92AaeXatLxBI9gBaebbnrfifHhDYfgasaacH8akY=wiFfYdH8Gipec8Eeeu0xXdbba9frFj0=OqFfea0dXdd9vqai=hGuQ8kuc9pgc9s8qqaq=dirpe0xb9q8qiLsFr0=vr0=vr0dc8meaabaqaciaacaGaaeqabaqabeGadaaakeaaiiGacqWFdpWCdaqhaaWcbaGaemyAaKgabaGaeGOmaidaaaaa@30F0@/*n*, and we interpret the shift parameter *δ *relative to *r*_*i*_. The units of *r*_*i *_are not very interpretable, however, so we use of pilot data as a guide. That is for example, if in our pilot data the genes that we call significant have |*r*_*i*_| > 100, we can set *δ *= 100 in our sample size assessment.

### Remark

In the SAM approach, the denominator *s*_*i *_in the score (1) is replaced by *s*_*i *_+ *s*_0_, where *s*_0 _is an exchangeability constant. It shrinks the scores of genes with expression near 0 (having *s*_0 _≈ 0).

## An example

**Figure 1:**
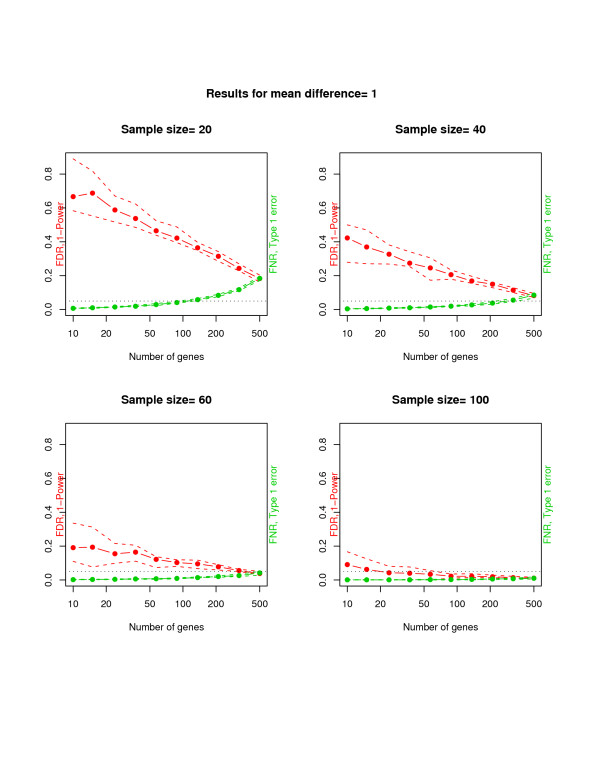
Results for simulated data. The genes are generated independently. Each panel shows the estimated FDR and FNR (solid red and green curves) as well as the 10 and 90th percentiles, using the proposed method (remember that in our setup FDR = 1-power and FNR = type I error). A horizontal line is drawn at 0.05. The quantity on the horizontal axis – **number of genes **– refers to both the hypothesized number of truly non-null genes, and the number of genes called significant. We see that the FDR is probably too high for the pilot data sample size of 20, but improves considerably when the sample size is doubled to 40.

We generated some pilot data in two classes: there were a total of 1000 genes and 20 samples, with 10 samples in each of class. Each measurement was standard Gaussian (i.e. there was no difference between the groups in the pilot data). We ran a SAM permutation analysis, assuming the data are in a log base 2 scale and specifying a mean difference of Iog_2 _2 = 1.0. This corresponds to a mean difference of 2 fold for class 1 versus class 2. The results are shown in Figure 1.

Remember that the quantity on the horizontal axis – **number of genes **– refers to both the hypothesized number of truly non-null genes, and the number of genes called significant.

We see that, depending on the number of genes truly changed at 2-fold, the sample size should be increased to 60 or 100, in order to get the FDR down to 10% or 5%. The false negative rate is consistently low throughout, when *n *= 60 or 100.

**Figure 2:**
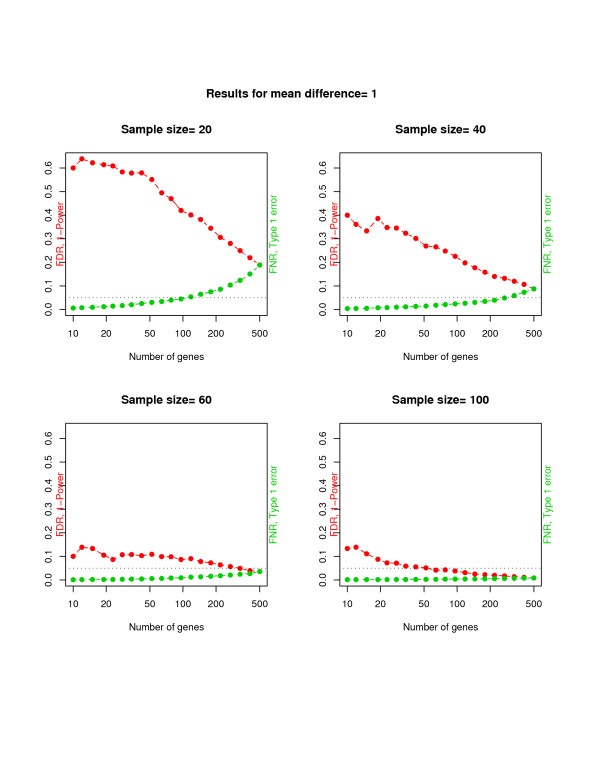
Results for first simulation study. Here the FDR and FNR are estimated by direct simulation from underlying model.

Does our approach provide accurate estimates of FDR and FNR? For the setup of the previous example, we estimated FDR and FNR directly from repeated simulations of data from the underlying model. The results are shown in Figure 2.

Note the similarity between Figures 1 and 2. Of course with real data, the second method – generating data from the underlying model – would not be available, since the underlying model is unknown.

**Figure 3:**
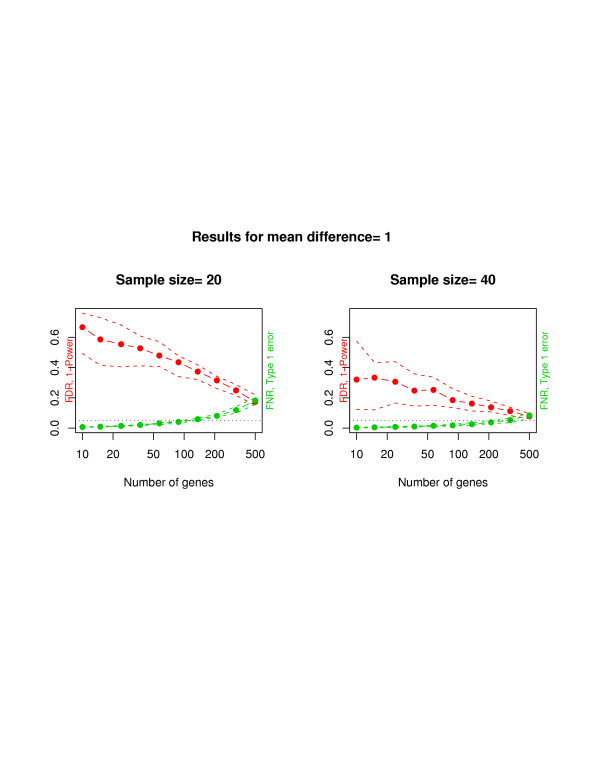
Results for second simulated example (correlated genes).

Figure 3 shows a second example. Here there are 20 samples, and 10 blocks of 100 genes, with genes having pairwise correlation 0.5 in in each block. The mean structure is the same as in the previous example. We see that the FDR and FNR curves are similar to those in Figure 1, but the 10% and 90% curves are much wider. With less certainty in the estimate, it would be advisable to take a large sample size to ensure a reasonably low FDR. This illustrates the importance of preserving the correlation structure of the genes, i.e. it is not safe to make the (unrealistic) assumption of independence between the genes.

## Discussion

We have presented a simple method for assessing sample sizes, that starts with a permutation-based analysis for some pilot data. The method gives reasonably accurate estimates of false discovery rates and false negative rates, as a function of the total number of samples. Our proposal is implemented in the SAM package- the Excel add-in and the R package **samr **[1].

## Figures and Tables

**Table 1:** Possible outcomes from m hypothesis tests of a set of genes. The rows represent the true state of the population and the columns are the result a data-based decision rule.

	Called Not Significant	Called Significant	Total
Null	*U*	*V*	*m*_0_
Non-null	*T*	*S*	*m*_1_
Total	*m – R*	*R*	*m*
